# Simulation of the inelastic deformation of porous reservoirs under cyclic loading relevant for underground hydrogen storage

**DOI:** 10.1038/s41598-022-25715-z

**Published:** 2022-12-10

**Authors:** Kishan Ramesh Kumar, Herminio Tasinafo Honorio, Hadi Hajibeygi

**Affiliations:** Faculty of Civil Engineering and GeoSciences, Delft University of Technology, 2628 CD Delft, The Netherlands

**Keywords:** Solid Earth sciences, Petrology, Computational science

## Abstract

Subsurface geological formations can be utilized to safely store large-scale (TWh) renewable energy in the form of green gases such as hydrogen. Successful implementation of this technology involves estimating feasible storage sites, including rigorous mechanical safety analyses. Geological formations are often highly heterogeneous and entail complex nonlinear inelastic rock deformation physics when utilized for cyclic energy storage. In this work, we present a novel scalable computational framework to analyse the impact of nonlinear deformation of porous reservoirs under cyclic loading. The proposed methodology includes three different time-dependent nonlinear constitutive models to appropriately describe the behavior of sandstone, shale rock and salt rock. These constitutive models are studied and benchmarked against both numerical and experimental results in the literature. An implicit time-integration scheme is developed to preserve the stability of the simulation. In order to ensure its scalability, the numerical strategy adopts a multiscale finite element formulation, in which coarse scale systems with locally-computed basis functions are constructed and solved. Further, the effect of heterogeneity on the results and estimation of deformation is analyzed. Lastly, the Bergermeer test case—an active Dutch natural gas storage field—is studied to investigate the influence of inelastic deformation on the uplift caused by cyclic injection and production of gas. The present study shows acceptable subsidence predictions in this field-scale test, once the properties of the finite element representative elementary volumes are tuned with the experimental data.

## Introduction

One of the significant threats facing our planet is climate change caused mainly by emissions from fossil fuels. To address this problem, the world is increasingly drifting towards renewable energy sources, such as solar and wind. However, the main drawback of renewable energy is its intermittent nature due to the fluctuating seasonal events like wind forces^1^. This results in excess and deficits of renewable energy production, while consumption graphs have much more steady patterns. Therefore, feasible large-scale energy storage—in the order of TWh—technologies are needed in order to ensure that the generated excess power can be used at times of under production. One option is to convert the renewable energy to hydrogen through electrolysis^2^ and further store it inside depleted oil and gas fields, or salt caverns^3,4^. Note that hydrogen demand is increasing in many areas such as in heavy-duty transportation, chemical and steel industries^5–7^. Due to the seasonally-intermittent nature of the renewable energy production, the reservoirs would undergo cyclic loading of injection/production of hydrogen. Subsurface storage technology has been implemented already by storing e.g. hydrogen in salt caverns at Teesside (UK) and the US Gulf coast^8^. A list of all active projects on underground hydrogen storage (UHS) can be found in the literature^9^. Natural gas storage is much more common across the world, including e.g. the four sites in the Netherlands^10,11^. For safe operations, it is critical to quantify the impact of altering the reservoir pressure on the rock deformation and state of stress^12,13^. This quantification needs to be based on appropriate nonlinear deformation physics, specially viscoplasticity, when the stress field crosses the yield tolerance limits of the rock elements.

Microbial activity, geochemistry, geomechanics, and hydrogeology (multi-phase flow) are the four critical aspects of hydrogen storage that need to be addressed individually and simultaneously to understand their impact on energy storage^14^. This work focuses on geomechanics and calibrating its risks, for example, regarding surface subsidence/uplift^15,16^. Due to cyclic injection and production, the reservoir undergoes plastic deformation, which becomes critical due to the presence of lots of heterogeneities such as faults and fractures, especially over long operational periods. Since seasonal storage projects are the focus of the UHS and natural gas storage projects, the numerical experiments need to allow for simulation of the deformation over long periods (several years). Analysis of the rock stress and deformation over such long time scales helps to reveal potential risks associated with this storage process, for instance, an initially stable storage platform can indeed become critically stressed after a long operational time^17,18^.

One of the major concerns of energy storage in the subsurface is the consequent ground surface subsidence or uplift. Several researchers in the past have reported subsidence in the carboniferous sandstone fields across the world which are used to produce hydrocarbons^17,19–24^. When these reservoirs are used to store hydrogen, this results in seasonal cyclic loading on the reservoir that could cause permanent subsidence or uplift depending on the operating conditions and the rock characteristics. This calls for accurate modelling of subsidence for understanding the grain scale physics of the rocks in the subsurface. Some researchers modify the variation of elastic properties along depth and study the subsidence based on elasticity and pore-elasticity^25–27^. However this approach ignores the underlying grain scale physics, especially in the long-term (years) of cyclic loading. The authors in^28^, for instance, investigate real case subsidence in the Bergermeer reservoir by developing a full scale 3D poromechanical model, which includes a viscoelastic model (Kelvin–Voigt), faults and fractures. The effects of inelastic deformations were thus neglected from their work. Many underground reservoirs (and their surroundings), however, are composed of different types of rocks with complex and particular mechanical behaviors. Inelastic deformations such as plasticity, viscoplasticity and creep are commonly observed in underground formations. Plasticity is a permanent deformation that occurs instantaneously (time-independent) when the stress levels reach a certain yield limit (yield surface). Similarly, viscoplasticity also refers to a permanent deformation when the stress levels touch the yield surface. In this case, however, the rate at which stress is applied also plays an important role^29^, and the stress levels are allowed to exceed the yield surface and then it takes a finite amount of time for it to return to the yield surface. Therefore, unlike plasticity, viscoplasticity is a time-dependent inelastic deformation. Another type of time-dependent inelastic deformation is creep, in which the material constantly deforms under the application of a constant and persistent external load, irrespective of the stress levels.

Although it is not possible to experimentally distinguish between creep and viscoplasticity^30^, they actually follow different types of physics and, consequently, have different constitutive laws. For instance, creep is often modeled using power-laws^31–33^, although more general formulations are also available in the literature^34–36^. Several experiments have been conducted in the past to study creep deformations in rocks^37–42^. Viscoplastic models for rock/soil have also been proposed by several researchers^43–46^. Some of them also use viscoplastic formulations to model creep deformations^46,47^, since viscoplastic deformations tend to creep deformations when the yield limit tends to zero^30^. For modeling plasticity (and also viscoplasticity), the critical state theory (CST) has been long employed to describe inelastic deformation in a variety of weak rocks including chalk, bonded mudrocks, carbonates, sandstones and sand^48–51^. The modified cam-clay model (MCC), as introduced by^52^, is the most widely used critical state model, which incorporates imposed loading sensitivity and hardening or softening hypothesis. Many researchers^53,54^ have shown the applicability of modified cam clay plasticity model in the computation of inelastic deformation of sandstone rocks which is caused due to inter-granular cracking and slip of inter-granular clay films. Considering this hypothesis, MCC model is used in our work although the extent to which plasticity models can effectively describe inelastic deformation in the reservoir scale is still under research. An aspect of rock/soil failure analysis relates to the impact of cyclic loading, especially for intermittent injection/withdrawal of underground reservoirs. In this case, the material might undergo plastic deformation sooner than normally expected. A few studies can be mentioned in which the researchers analyze the effect of cyclic loading on rocks^55–58^ using experiments under different confining pressures, loading conditions and chemical environments.

The first goal of this work is to propose a numerical model to properly describe the mechanical behavior of an underground formation composed of materials with different inelastic behaviors. A highly permeable sandstone reservoir is considered to be surrounded by a sandstone formation with low permeability, shale rock and salt rock. For salt rock, only creep deformation is accounted. Creep is also considered for the sandstone formation, as well as plastic deformations. Finally, viscoplascitiy is incorporated for the shale rock model. Power-law^44^ is employed for modelling creep in both salt rock and sandstone. The sandstone plasticity is modeled by using the MCC as a yield criterion and the consistency algorithm, as presented in^50^. In order to account for cyclic loading effects, we employ the strategy proposed by^59^, in which the equation for the hardening parameter update is properly modified. Recently, cyclic experiments on sandstone have been conducted^60^, where a cyclic MCC model was used to match the experimental results. Finally, the viscoplastic response of shale rock is modeled through Perzyna’s^46^ algorithm and the MCC as a yield criterion. A detailed description of how these models can be combined is provided within this work.

In addition to the complex mechanical behaviors, underground formations are often highly heterogeneous, which poses a serious challenge from a numerical point of view. The reason for this is that extremely fine grids are often necessary to properly capture such highly heterogeneous mechanical properties. As a consequence, the resulting system of equations is excessively large, thus requiring prohibitively computational power. In this context, a second contribution of this work is to devise a multiscale computational framework to study cyclic loading effects on reservoir rocks undergoing different types of inelastic strains, namely creep, viscoplasticity and plasticity. First, a fine-scale consistent modeling approach, based on finite element discrete scheme, is developed. Further, the fine-scale computational framework is then extended to a multiscale framework allowing for representation of the computational system on a much coarser resolution.

Multiscale strategy has been implemented in the past for solving porous media flow in the subsurface^61–68^. This strategy has been implemented also for geo-mechanics^69–72^. The main idea of the multiscale strategy is solving the system at coarse scale and further approximating the solution to the finescale. Development of the multiscale strategy is not only important for advancing the computational efficiency, as formerly addressed for linear mechanics but also crucial to allow for generating a consistent map between fine and coarse scale systems without any need of upscaled parameters. This can also allow for the connection of the coarse scale observation data directly to the fine-scale computational system, to reduce the uncertainty, as shown for flow in porous media^73^. For convenient integration within a given simulation framework, the developed multiscale strategy is also formulated and implemented algebraically.

Numerical results are presented to study various aspects of the developed computational strategy. Firstly, the developed fine-scale finite-element method (FEM) simulation method is benchmarked with the literature. Additionally, the impact of inelastic deformation in a field-scale relevant test case is studied. It is worth mentioning that an important consequence of altering the subsurface stress is the subsidence/uplift; specially if the site is close to populated urban areas^74–76^. In this study, as a relevant test case, the Dutch Bergermeer gas field is also investigated in detail to quantify the associated uplift over a few years of cyclic loading.

The paper is structured as follows. The governing equations related to solid mechanics are presented in “Governing equations”. The inelastic deformations, namely creep, viscoplasticity and plasticity, as well as the main ingredients of the MCC model, are discussed in “ Inelastic deformations”. In “Numerical formulation”, in addition to the finite element formulation, the implementation of the combined inelastic models is described in details. Once the discretized equations are obtained for the fine scale grid, the multiscale (MS) strategy is employed in “Multiscale formulation” to obtain the coarse grid algebraic equations. Finally, numerical experiments are carried out in “Results”, after which the conclusions are drawn in “Conclusions”.

## Governing equations

A rock undergoing an external load can be represented by the linear momentum conservation equation^77^. Assuming quasi–static loading condition (neglecting inertial effects), this equation can be expressed as

1 $$\begin{aligned} \nabla \cdot {\varvec{\sigma }} = -{\textbf{f}}, \end{aligned}$$

where ${\varvec{\sigma }}$ is the rank-2 stress tensor and ${\textbf{f}}$ denotes the volumetric body forces, expressed as (N/m$^3$). Moreover, the stress tensor can be expressed in terms of the rank-4 stiffness tensor ${\mathbb {C}}$ and the rank-2 elastic strain tensor ${\varvec{\varepsilon }}^e$, that is,

2 $$\begin{aligned} {\varvec{\sigma }} = {\mathbb {C}}:{\varvec{\varepsilon }}^e. \end{aligned}$$

For isotropic linear elastic materials, according to Hooke’s law the elements of the stiffness tensor are given by

3 $$\begin{aligned} {\mathbb {C}}_{ijkl} = \lambda \delta _{ij}\delta _{kl} + G(\delta _{ik}\delta _{jl} + \delta _{il}\delta _{jk}), \end{aligned}$$

where $\delta _{ij}$ is the Kronecker’s delta and $\lambda$ and *G* are the first Lame’s parameter and shear modulus, respectively.

When the small strain assumption is valid, the total strain tensor ${\varvec{\varepsilon }}$ has a linear relation with the displacement vector ${\textbf{u}}$, given by,

4 $$\begin{aligned} {\varvec{\varepsilon }} = \nabla _s {\textbf{u}} \equiv \frac{1}{2} \left( \nabla {\textbf{u}} + \nabla {\textbf{u}}^T \right) , \end{aligned}$$

with $\nabla _s$ denoting the symmetric nabla operator. Additionally, the small strain assumption allows the total strain tensor to be split into an elastic (${\varvec{\varepsilon }}^e$) and an inelastic (${\varvec{\varepsilon }}^{ie}$) parts, that is,

5 $$\begin{aligned} {\varvec{\varepsilon }} = {\varvec{\varepsilon }}^e + {\varvec{\varepsilon }}^{ie}. \end{aligned}$$

Combining Eqs. (1), (2) and (4), the linear momentum balance equation can be expressed as

6 $$\begin{aligned} \nabla \cdot \left( {\mathbb {C}}:{\varvec{\varepsilon }} \right) = -{\textbf{f}} + \nabla \cdot \left( {\mathbb {C}}:{\varvec{\varepsilon }}^{ie} \right) . \end{aligned}$$

One of the challenges here is to properly account for the different types of inelastic deformations of shale, salt and sandstone formations. In the next section, the inelastic deformations considered in this work are presented in detail.

## Inelastic deformations

The rock formations considered in this work are known to present time-dependent and time-independent inelastic deformations. Time-independent inelastic deformation basically refer to plastic deformations, which occur when the effective stress reach a certain point. Similarly, viscoplasticity consists of irreversible deformations that develop over time, regarded that the stress levels also reach a critical value. The phenomenon known as creep also refer to a time-dependent irreversible deformation, however, it occurs for any level of stress.

In this work, only creep is considered for the inelastic deformation of salt rock. Creep is also considered for the sandstone, in addition to plastic (time-independent) deformations. Finally, for shale rocks only viscoplastic deformations are considered. The Modified Cam-Clay (MCC) failure criteria adapted for cyclic loading condition is adopted for both shale rock and sandstone. For shale rock, however, the Perzyna algorithm, which adopts the concept of overstress, is employed. On the other hand, Consistency based algorithm is used for sandstone. In what follows, the creep model, the MCC, Perzyna and Consistency algorithms are briefly presented. Associative plasticity model is employed in this work.

### Creep

**Figure 1:**
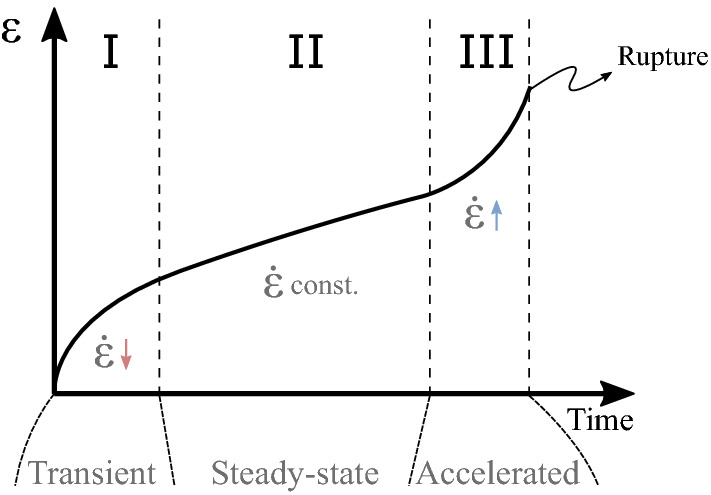
Creep deformation curve under constant external load.

Creep deformation is a well-known phenomenon observed in a broad range of materials. In the present case, this deformation mechanism can be observed in both rock salt and sandstone. As illustrated in Fig. 1, a typical creep curve is characterized by three distinct regions: transient (I), steady-state (II) and accelerated (III) creep. The visco-elastic response of the transient creep often occurs in a very short period of time when compared to the steady-state creep. Also, for short periods of time, the accumulated strain for stages I and II can be of the same order of magnitude. However, for sufficiently long periods of time the amount of strain accumulated during stage I can be negligible when compared to stage II. Considering all uncertainties associated with the geological data, it is reasonably safe to omit this initial creep stage. Predicting material rupture is not the purpose of this work, hence the accelerated creep stage is also disregarded.

In the presence of a constant external load, the steady-state creep is characterized by a constant strain rate, as shown in Fig. 1. The strain rate caused by steady-state creep can be modeled by^44^

7 $$\begin{aligned} \dot{{\varvec{\varepsilon }}}^{cr} = A \exp \left( -\frac{Q}{RT}\right) q^{n-1} {\textbf{s}} \end{aligned}$$

where *Q* is the activation energy, *R* is Boltzmann’s constant and *T* is temperature. Finally, *A* and *n* are material constants and *q* is the von Misses stress, given by $q=\sqrt{\frac{2}{3} {\textbf{s}}:{\textbf{s}}}$, with ${\textbf{s}}$ being the deviatoric stress tensor.

Several researchers have shown that the rocksalt undergoes creep^33,78,79^. In this work, only the steady state creep is considered for rock using a power law as shown in Eq. (7). Similarly, few researchers have also shown that the sandstone rock undergoes creep^37,42,80^. In this work, creep in sandstone rocks is also accommodated using the power law formulation, as shown in Eq. (7).

### Modified Cam-Clay (MCC)

The basic steps involved in the Modified Cam-Clay model are presented in this section. The MCC is employed in this work for both plasticity and visco-plasticity models. For the purpose of keeping the notation simple, we adopt the superscript $``p''$ throughout this section to denote either *plastic* or *visco-plastic* deformations. In “Perzyna based viscoplastic algorithm” and “Consistency based plastic algorithm”, as well as the remaining sections, however, the superscripts $``p''$ and $``vp''$ are used to properly differentiate between plastic and visco-plastic deformations, respectively.

**Figure 2:**
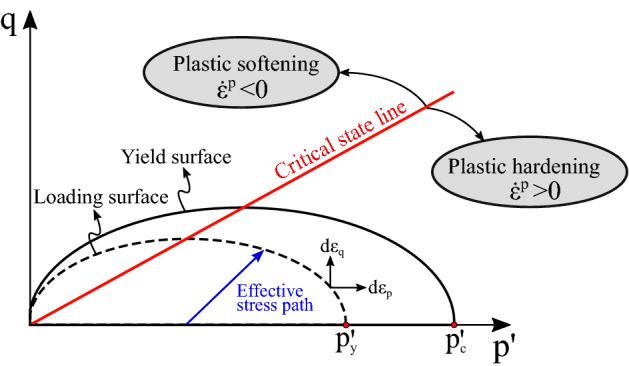
The illustration shows the schematic of the MCC model in the stress space. It indicates how the yield surface develops, depending on the effective stress path, highlighting the plastic hardening (below the CSL) and the plastic softening region (above the CSL).

Based on loading conditions and material properties, a certain yield function is responsible for deciding whether the material deformation is under elastic or (visco)plastic regime. The main ingredients of the MCC are the (1) elastic step, (2) yield function, (3) the hardening parameter and (4) plastic flow rule^46,50,81,82^. The schematic representation of the MCC model is shown in Fig. 2. The elastic region is enclosed by the yield surface represented by the semi-ellipse. The figure shows the yield surface, in solid black line, and a “loading” surface, represented by the dashed black line, both of which are discussed below. The effective stress path is characterized by the load applied to the rock, and it is represented in the $p'$–*q* plane, with $p'$ being the mean volumetric stress ($p'=-\textrm{tr}({\varvec{\sigma }})/3$) and *q* representing the von Misses stress. Moreover, the effective stress path for the drained test case dictates whether the material undergoes plastic hardening or plastic softening. The Critical State Line (CSL) shows the terminal state of the rock in the plastic region. A material yielding at the critical state does not compact or dilate when sheared. In other words, the CSL separates the dilation and compression regions of the rock. In what follows, the four ingredients mentioned at the beginning of this paragraph are presented in more detail.

#### Elastic step

In soil mechanics, it can be observed experimentally that the void ratio (*e*) has a non-linear relationship with the mean stress ($p'$), which is given by

8 $$\begin{aligned} e = C - \kappa \ln p', \end{aligned}$$

where $\kappa$ is the compression index and *C* is a material constant corresponding to $\ln (p'=1)$. Additionally, the change in the void ratio is given by

9 $$\begin{aligned} \textrm{d} e = -(1+e_0) \textrm{d} \varepsilon _v, \end{aligned}$$

with $\textrm{d}{\varepsilon }_v$ representing the incremental volumetric strain and $e_0$ denoting the initial void ratio.

The void ratio is a measure of how tight the soil grains are to each other. It is reasonable to assume that the more connected the soil grains are to each other, the larger the material stiffness is. In fact, from classical soil mechanics, it can be shown that the relationship between the void ratio and the elastic moduli are

10 $$\begin{aligned} K = \frac{(1+e)p'}{\kappa } \quad \textrm{and} \quad G = \frac{3(1-2\nu )(1+e)p'}{2(1+\nu )\kappa }, \end{aligned}$$

with $\nu$ being the Poisson’s ratio. From Eq. (10) it can be noticed that the elastic step in the MCC model is actually non-linear, since the elastic moduli also change with both mean stress $p'$ and void ratio *e*.

#### Yield function

The yield function refers to the equation that describes the yield surface, as depicted in Fig. 2. The yield function ${\mathscr {F}}$ depends on the stress level and the (visco)plastic strain, and it can be expressed as

11 $$\begin{aligned} {\mathscr {F}} ({\varvec{\sigma }}, \varepsilon _v^{p}) = q^2 + M^2p'(p' - p'_c(\varepsilon _v^{p}))=0, \end{aligned}$$

where *M* is the slope of the CSL and $p'_c$ is the hardening parameter, which is also called as the pre-consolidation pressure and it depends on the volumetric (visco)plastic strain $\varepsilon _v^{p}$. As shown in Fig. 2, the position of $p'_c$ on the $p'$ axis changes according to the yield surface ${\mathscr {F}}$.

Instead of evaluating the yield function (Eq. 11), it can be also convenient to define a semi-elliptical “loading” surface, as represented by the dashed black line in Fig. 2. The point $p_y'$, which intersects the $p'$ axis, is given by

12 $$\begin{aligned} p_y' = p' + \left( \frac{q}{M}\right) ^2\frac{1}{p'}. \end{aligned}$$

In this manner, a simple comparison between $p'_c$ and $p'_y$ is enough to characterize whether the material behaves elastically ($p_y'<p_c'$) or plastically ($p_y'=p_c'$).

#### Hardening parameter

The hardening parameter is characterized by the variable $p_c'$, which defines the position of the yield surface. Therefore, the material becomes “harder” when $p_c'$ increases, since a higher stress level must be achieved to trigger plastic deformations. In the original approach of the MCC model, the hardening parameter increment (d$p_c'$) is zero if the stress state is in the elastic regime, for instance $p_y'<p_c'$. Otherwise, if the stress state touches the yield surface ($p_y'=p_c'$), then the hardening parameter increases proportionally to the (visco)plastic strain variation. This can be expressed as follows.

13 $$\begin{aligned} \frac{\textrm{d} p'_c}{p'_c} = {\left\{ \begin{array}{ll} 0 &{} \text {if}\, p_y'<p_c', \\ \left( \dfrac{1+e}{\lambda - \kappa }\right) \textrm{d} \varepsilon _v^{p} &{} \text {otherwise.} \end{array}\right. } \end{aligned}$$

where $\lambda$ is the unloading/reloading index, which is obtained from isotropic plastic consolidation test^83^.

The hardening rule represented by Eq. (13) is originally developed for monotonic loads. For cyclic loads, however, even in the elastic region, the material might reach failure sooner than predicted by the original MCC. This suggests that the yield surface actually shrinks during the process. Based on this idea, the hardening rule represented by Eq. (13) has been generalized for cyclic loading^59^ by allowing the yield surface to shrink during unloading (d$p_y'<0$). This case can be expressed as

14 $$\begin{aligned} \frac{\textrm{d} p'_c}{p'_c} = {\left\{ \begin{array}{ll} 0 &{} \text {if}\, p_y'<p_c'\, \hbox {and} \,\hbox {d}\,p_y'\ge 0, \\ \theta \dfrac{\textrm{d} p'_y}{p'_y} &{} \text {if }\,p_y'<p_c'\, \hbox {and} \,\hbox {d}\,p_y'<0, \\ \left( \dfrac{1+e}{\lambda - \kappa }\right) \textrm{d} \varepsilon _v^{p} &{} \text {otherwise.} \end{array}\right. } \end{aligned}$$

in which $\theta$ represents the fraction of shrinkage of the yield surface with respect to the “loading” surface. Notice that Eq. (13) is fully recovered from Eq. (14) when $\theta =0$. In a recent work^60^, the authors conducted cyclic triaxial experiments on sandstone rock and observed similar trend of the applicability of $\theta$ parameter in sandstone experiments.

#### Plastic flow rule

Plastic strain is computed using the plastic flow rule, which is dependent on the stresses. In this work, we employ an associative flow rule, in which the plastic (or viscoplastic, in this case) strain is proportional to the gradient of the yield function with respect to stress. The incremental (visco)plastic strain tensor and the incremental volumetric (visco)plastic strain, therefore, take the following form

15 $$\begin{aligned} \textrm{d} {\varvec{\varepsilon }}^{p} = \gamma \frac{\partial {\mathscr {F}} }{\partial {\varvec{\sigma }}} \quad \text {and} \quad \textrm{d} \varepsilon ^{p}_v = \gamma \frac{\partial {\mathscr {F}} }{\partial p'} \end{aligned}$$

with $\gamma$ denoting the plastic multiplier. The definition of this parameter depends on the numerical strategy adopted. In this work, Perzyna and Consistency based algorithms are employed for this purpose.

The four main steps of a plasticity model have been presented above. In the remaining of this section, we present the numerical approaches employed for obtaining the plastic multiplier of Eq. (15).

#### Perzyna based viscoplastic algorithm

In this work, we follow the approach proposed in^46^, in which the viscoplastic strain rate tensor and viscoplastic volumetric strain rate are respectively given by,

16 $$\begin{aligned} \dot{{\varvec{\varepsilon }}}^{vp} = \dot{\gamma } \frac{ \partial _{{\varvec{\sigma }}} {\mathscr {F}} }{ |\partial _{{\varvec{\sigma }}} {\mathscr {F}}| } \quad \text {and} \quad \dot{\varepsilon }^{vp}_v = \dot{\gamma } \frac{ \partial _{p'} {\mathscr {F}} }{ |\partial _{{\varvec{\sigma }}} {\mathscr {F}}| }, \end{aligned}$$

where $\partial _{{\varvec{\sigma }}} {\mathscr {F}}$ and $\partial _{p'} {\mathscr {F}}$ are the partial derivatives of ${\mathscr {F}}$ with respect to ${\varvec{\sigma }}$ and $p'$, respectively. Moreover, the plastic multiplier is computed as,

17 $$\begin{aligned} \dot{\gamma } = \frac{\langle {\mathscr {F}}({\varvec{\sigma }}, \varepsilon _v^{vp}) \rangle }{\mu (\varepsilon _v^{vp}) p'} \end{aligned}$$

with $\langle \cdot \rangle$ representing the Macaulay brackets and the viscosity expressed as

18 $$\begin{aligned} \mu (\varepsilon _v^{vp}) = \mu _0 \exp \left( \zeta \varepsilon _v^{vp} \right) \end{aligned}$$

in which $\mu _0$ and $\zeta$ are the reference viscosity and a material parameter.

Defining the residues

19 $$\begin{aligned} \psi&= \frac{{\mathscr {F}}}{p'} - \dot{\gamma } \mu \left( \varepsilon _v^{vp} \right) , \end{aligned}$$

20 $$\begin{aligned} {\textbf{r}}&= - \text {d} {\varvec{\varepsilon }} + \text {d} {\varvec{\varepsilon }}^e + \text {d} {\varvec{\varepsilon }}^{vp}, \end{aligned}$$

where Eq. (19) is the residual form of Eqs. (17) and (20) neglects creep and plasticity contributions, since the Perzyna algorithm is employed here only for the shale rock, where creep and time-independent plasticity are assumed to be absent. Equations (19) and (20) depend on ${\varvec{\sigma }}$ and $\dot{\gamma }$, therefore, applying Newton‘s method and solving for the increments d${\varvec{\sigma }}$ and d$\dot{\gamma }$ leads to

21 $$\begin{aligned} \text {d}\dot{\gamma }&= \frac{\psi - \partial _{{\varvec{\sigma }}}\psi :\partial _{{\varvec{\sigma }}}{\textbf{r}}^{-1}:{\textbf{r}}}{\partial _{{\varvec{\sigma }}}\psi :\partial _{{\varvec{\sigma }}}{\textbf{r}}^{-1}:\partial _{\dot{\gamma }}{\textbf{r}}}, \end{aligned}$$

22 $$\begin{aligned} \text {d}{\varvec{\sigma }}&= - \partial _{{\varvec{\sigma }}}{\textbf{r}}^{-1}:\left( {\textbf{r}} + \partial _{\dot{\gamma }} {\textbf{r}} \text {d}\dot{\gamma } \right) . \end{aligned}$$

The reader is referred to^46^ for the detailed formulation, including the explicit expressions for $\partial _{{\varvec{\sigma }}}\psi$, $\partial _{\dot{\gamma }}\psi$, $\partial _{{\varvec{\sigma }}}{\textbf{r}}$ and $\partial _{\dot{\gamma }}{\textbf{r}}$. Therefore, for each time step, internal iterations are required by updating stress and plastic multiplier as $\dot{\gamma }^{k+1} = \dot{\gamma }^k + \text {d}\dot{\gamma }$ and ${\varvec{\sigma }}^{k+1} = {\varvec{\sigma }}^{k} + \text {d} {\varvec{\sigma }}$.

#### Consistency based plastic algorithm

For the sandstone, plasticity is incorporated through the consistency algorithm in conjunction with the MCC model, with the hardening parameter also computed by Eq. (14). Plastic deformation occurs when the yield function value is equal to 0. The return mapping algorithm using consistency condition has been used by several researchers^50,81,82,84^. We adopt the approach suggested by Liu et al.^50^ in which anisotropy is ignored. Here, the plastic multiplier is computed by assuming the consistency condition, given by

23 $$\begin{aligned} \text {d}{\mathscr {F}} = \frac{\partial {\mathscr {F}}}{\partial {\varvec{\sigma }}}:\text {d}{\varvec{\sigma }} + \frac{\partial {\mathscr {F}}}{\partial p_c'} \text {d}p_c' = 0. \end{aligned}$$

The increment of stress, d${\varvec{\sigma }}$, is proportional to the elastic strain. Since sandstone is considered in this work to undergo elastic, creep and plastic deformations, the increment of stress can be expressed as

24 $$\begin{aligned} \text {d}{\varvec{\sigma }} = {\mathbb {C}}:\left( \text {d}{\varvec{\varepsilon }} - \text {d}{\varvec{\varepsilon }}^{cr} - \text {d}{\varvec{\varepsilon }}^{p} \right) \end{aligned}$$

with $\text {d}{\varvec{\varepsilon }}$ being the increment of total deformation. The increment of plastic deformation can be obtained from the plastic flow rule (Eq. 15). Additionally, Eq. (14) for the case of $p_y'\ge p_c'$ can be solved for the increment d$p_c'$. In this manner, Eq. (23) becomes

25 $$\begin{aligned} \frac{\partial {\mathscr {F}}}{\partial {\varvec{\sigma }}} : {\mathbb {C}}:\left( \text {d}{\varvec{\varepsilon }} - \text {d}{\varvec{\varepsilon }}^{cr} \right) - \frac{\partial {\mathscr {F}}}{\partial {\varvec{\sigma }}} : {\mathbb {C}}: \gamma \frac{\partial {\mathscr {F}}}{\partial {\varvec{\sigma }}} + \gamma \frac{\partial {\mathscr {F}}}{\partial p_c'} \frac{\partial {\mathscr {F}}}{\partial p'} \left( \dfrac{1+e}{\lambda - \kappa }\right) p_c' = 0 \end{aligned}$$

Finally, solving Eq. (25) for $\gamma$ provides

26 $$\begin{aligned} \gamma = \dfrac{ \frac{\partial {\mathscr {F}}}{\partial {\varvec{\sigma }}} : {\mathbb {C}}:\left( \text {d}{\varvec{\varepsilon }} - \text {d}{\varvec{\varepsilon }}^{cr} \right) }{ \frac{\partial {\mathscr {F}}}{\partial {\varvec{\sigma }}} : {\mathbb {C}}: \frac{\partial {\mathscr {F}}}{\partial {\varvec{\sigma }}} - \frac{\partial {\mathscr {F}}}{\partial p'_c} \frac{\partial {\mathscr {F}}}{\partial p'} \left( \dfrac{1+e}{\lambda - \kappa }\right) p_c' } \end{aligned}$$

The terms on the right-hand side of Eq. (26) must be evaluated at the current time level. For each time step, an iterative procedure (return mapping strategy) is employed on the stress until the corrected stress and the hardening parameter satisfy the yield condition.

**Figure 3:**
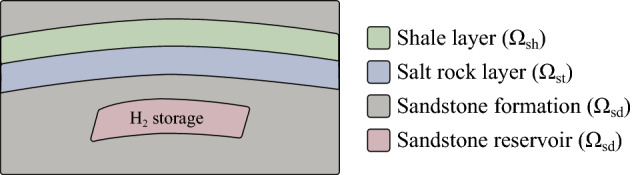
Schematic representation of the solution domain.

### Inelastic strain composition

As depicted in Fig. 3, the domain $\Omega$ is decomposed in $\Omega _\text {st}$, $\Omega _\text {sd}$ and $\Omega _\text {sh}$ for salt rock, sandstone and shale rock, respectively. Considering these different domains do not overlap each other, we have that

27 $$\begin{aligned} \Omega&= \Omega _\text {st} \cup \Omega _\text {sd} \cup \Omega _\text {sh}, \quad \text {and} \end{aligned}$$

28 $$\begin{aligned} \Omega _\text {st} \cap \Omega _\text {sd}&= \Omega _\text {st} \cap \Omega _\text {sh} = \Omega _\text {sd} \cap \Omega _\text {sh} = \emptyset . \end{aligned}$$

Considering these three materials, we propose to express the inelastic strain tensor of Eq. (6) as

29 $$\begin{aligned} {\varvec{\varepsilon }}^\text {ie} = {\varvec{\varepsilon }}_\text {st} + {\varvec{\varepsilon }}_\text {sh} + {\varvec{\varepsilon }}_\text {sd}, \end{aligned}$$

in which

30 $$\begin{aligned} {\varvec{\varepsilon }}_\text {m}({\textbf {x}}) = {\left\{ \begin{array}{ll} {\varvec{\varepsilon }}_\text {m}({\textbf {x}}) &{} \text {if } {\textbf {x}}\in \Omega _\text {m} \\ 0 &{} \text {otherwise} \end{array}\right. } \quad \text {for } \text {m} = \text {st}, \text {sd}, \text {sh} \end{aligned}$$

with the subscripts “st”, “sd” and “sh” denoting salt rock, sandstone and shale rock, respectively.

As mentioned before, the inelastic deformations are considered to be only creep for rock salt, creep and plasticity for sandstone and only viscoplasticity for shale rock. Therefore, the inelastic strain tensor can be further split into

31 $$\begin{aligned} {\varvec{\varepsilon }}^\text {ie} = {\varvec{\varepsilon }}_\text {st}^\text {cr} + {\varvec{\varepsilon }}_\text {sd}^\text {cr} + {\varvec{\varepsilon }}_\text {sd}^\text {p} + {\varvec{\varepsilon }}_\text {sh}^\text {vp}, \end{aligned}$$

where ${\varvec{\varepsilon }}_\text {st}^\text {cr}$ and ${\varvec{\varepsilon }}_\text {sd}^\text {cr}$ are modeled by Eq. (7), and plastic and viscoplastic strains are modeled using the MCC model with cyclic hardening parameter (Eq. 14). Moreover, the plastic multipliers $\gamma$ of ${\varvec{\varepsilon }}_\text {sd}^\text {p}$ and ${\varvec{\varepsilon }}_\text {sh}^\text {vp}$ are respectively obtained through the Perzyna and Consistency approaches previously discussed. We emphasize that all these inelastic strains must satisfy Eq. (30).

## Numerical formulation

Consider a domain $\Omega \in {\mathbb {R}}^2$ bounded by a surface $\Gamma$, which is oriented by a normal vector ${\textbf {n}}$ pointing outwards $\Omega$. The boundary surface can be further split in $\Gamma = \Gamma _D \cup \Gamma _N$, where $\Gamma _D$ and $\Gamma _N$ denote the portions of the boundary surface in which Dirichlet and Neumann boundary conditions are applied, respectively. Moreover, we have that $\Gamma _D \cap \Gamma _N = \emptyset$. The trial and test functions are respectively defined as

32 $$\begin{aligned} {\mathscr {U}}&= \lbrace {\textbf{u}} \in {\textbf{H}}^1, {\textbf{u}} = \bar{{\textbf{u}}} \text { on } \Gamma _D \rbrace , \end{aligned}$$

33 $$\begin{aligned} {\mathscr {W}}&= \lbrace {\textbf{w}} \in {\textbf{H}}^1, {\textbf{w}} = {\textbf{0}} \text { on } \Gamma _D \rbrace \end{aligned}$$

where ${\textbf{H}}^1$ denotes the first-order Sobolev space. In this manner, the weak form of Eq. (1) can be expressed as

34 $$\begin{aligned} \int _\Omega {\textbf {w}}^T(\nabla \cdot {\varvec{\sigma }} + {\textbf{f}}) \text {d}\Omega = 0, \quad \forall {\textbf {w}} \in {\mathscr {W}} \end{aligned}$$

The test function is assumed to be equal incremental solution (${\textbf{w}}=\delta {\textbf{a}}, \forall \delta {\textbf{a}} \in {\mathscr {W}}$) which satisfies the kinematic BC to minimize the residual in global domain. Using the divergence theorem,

35 $$\begin{aligned} \int _\Omega \delta {\textbf{a}}^T(\nabla \cdot {\varvec{\sigma }}) \text {d}\Omega = -\int _\Omega \delta {\varvec{\varepsilon }}^T {\varvec{\sigma }} dV + \int _\Gamma \delta {\textbf{a}}^T {\textbf {t}} \text {d}\Gamma \end{aligned}$$

where the imposed traction vector is given by ${\textbf {t}} = {\varvec{\sigma }} \cdot {\textbf {n}}$. Equation (35) is used in Eq. (34) to yield,

36 $$\begin{aligned} \int _\Omega \delta {\varvec{\varepsilon }}^T {\varvec{\sigma }} d\Omega = \int _\Gamma \delta {\textbf{a}}^T {\textbf {t}} d\Gamma + \int _\Omega \delta {\textbf{a}}^T {\textbf{f}} \text {d}\Omega . \end{aligned}$$

The above equation is the incremental internal virtual work which is equal to external virtual work. In Eq. (36), the first and second terms on the right-hand side represent the imposed boundary traction forces and the volumetric body forces, respectively. All these terms are evaluated at time $t+\Delta t$. In the incremental load/time-stepping scheme, the stress tensor can be written as

37 $$\begin{aligned} {\varvec{\sigma }}^{t+\Delta t} = {\varvec{\sigma }}^{t} + \Delta {\varvec{\sigma }}. \end{aligned}$$

Substituting Eq. (37) into Eq. (36),

38 $$\begin{aligned} \int _\Omega \delta {\varvec{\varepsilon }}^T \Delta {\varvec{\sigma }} \text {d}\Omega = \int _\Gamma \delta {\textbf{a}}^T {\textbf {t}} \text {d}\Gamma + \int _\Omega \delta {\textbf{a}}^T {\textbf {f}} \text {d}\Omega - \int _\Omega \delta {\varvec{\varepsilon }}^T {\varvec{\sigma }}^t \text {d}\Omega . \end{aligned}$$

By considering $\Omega ^h$ as a partition of $\Omega$ composed of non-overlapping quadrangles, the corresponding discrete function spaces ${\mathscr {U}}^h$ and ${\mathscr {W}}^h$ can be defined. Therefore, the displacement vector field can be approximated as

39 $$\begin{aligned} \delta {\textbf{a}}^T = \delta {\textbf{u}}^T \text {N}^T \quad \text {and} \quad \delta {\varvec{\varepsilon }}^T = \delta {\textbf{u}}^T \text {B}^T, \end{aligned}$$

with N and B representing the interpolation functions and their global derivatives, respectively. Accordingly, the discretized linear momentum equation can be written as,

40 $$\begin{aligned} \int _\Omega \delta {\textbf{u}}^T \text {B}^T \Delta {\varvec{{\varvec{\sigma }}}} d\Omega = \int _\Gamma \delta {\textbf{u}}^T \text {N}^T {\textbf {t}} d\Gamma + \int _\Omega \delta {\textbf{u}}^T\text {N}^T {\textbf {f}} d\Omega - \int _\Omega \delta {\textbf{u}}^T \text {B}^T {\varvec{\sigma }}^t d\Omega \end{aligned}$$

where $\delta {\textbf{u}}^T$ are constants and can be readily eliminated from Eq. (40). The incremental stress is proportional to the increment of elastic strain, that is

41 $$\begin{aligned} \Delta {\varvec{\sigma }} = {\mathbb {C}}\Delta {\varvec{\varepsilon }}^e, \end{aligned}$$

in which Voigt notation has been employed. However, considering infinitesimal strains, it follows that $\Delta {\varvec{\varepsilon }} = \Delta {\varvec{\varepsilon }}^e + \Delta {\varvec{\varepsilon }}^{ie}$ (from Eq. 5), and thus $\Delta {\varvec{\sigma }} = {\mathbb {C}} \left( \Delta {\varvec{\varepsilon }} - \Delta {\varvec{\varepsilon }}^{ie} \right)$. Additionally, recalling that $\Delta {\varvec{\varepsilon }} = \text {B} \Delta {\textbf {u}}$, then

42 $$\begin{aligned} \Delta {\varvec{\sigma }} = {\mathbb {C}} \left( \text {B} \Delta {\textbf {u}} - \Delta {\varvec{\varepsilon }}^{ie} \right) . \end{aligned}$$

Substituting Eq. (42) into Eq. (40),

43 $$\begin{aligned} \int _\Omega \text {B}^T {\mathbb {C}}\text {B}\Delta {\textbf{u}} d\Omega = \int _\Gamma \text {N}^T {\textbf{t}} \text {d}\Gamma + \int _\Omega \text {N}^T {\textbf{f}} \text {d}\Omega - \int _\Omega \text {B}^T {\varvec{\sigma }}^t \text {d}\Omega + \int _\Omega B^T {\mathbb {C}} \Delta {\varvec{\varepsilon }}^{ie} \text {d}\Omega . \end{aligned}$$

Equation (43) originates a system of equations in the following form

44 $$\begin{aligned} \text {K} \Delta {\textbf {u}} = {\textbf {b}}, \end{aligned}$$

where $${\textbf {b}} = {\textbf {b}}^e + {\textbf {b}}^{ie}$$, with

45 $$\begin{aligned} {\textbf {b}}^e = \int _\Gamma \text {N}^T {\textbf{t}} \text {d}\Gamma + \int _\Omega \text {N}^T {\textbf{f}} \text {d}\Omega - \int _\Omega \text {B}^T {\varvec{\sigma }}^t \text {d}\Omega \quad \text {and} \quad {\textbf {b}}^{ie} = \int _\Omega B^T {\mathbb {C}} \Delta {\varvec{\varepsilon }}^{ie} \text {d}\Omega . 
\end{aligned}$$

Because of the non-linearity present in ${\textbf {b}}^{ie}$, due to the inelastic strains, it is convenient to define a residue vector as

46 $$\begin{aligned} {\mathscr {R}} = {\textbf {b}} - \text {K} {\textbf {x}}, \end{aligned}$$

where ${\textbf {x}} = \Delta {\textbf {u}}$. Newton’s method can be applied by expanding Eq. (46) using Taylor series, that is,

47 $$\begin{aligned} {\mathscr {R}}^{k+1} = {\mathscr {R}}^k + \left. \frac{\partial {\mathscr {R}}}{\partial {\textbf{x}}} \right| ^k \Delta {\textbf{x}}. \end{aligned}$$

Enforcing the residue at the current iteration $k+1$ equal to zero, the following linearized system is repeatedly solved until convergence for each time level,

48 $$\begin{aligned} \text {J}^k \Delta {\textbf{x}} = - {\mathscr {R}}^k, \end{aligned}$$

with $\text {J}^k = \partial _{\textbf{x}} {\mathscr {R}}^k$ representing the Jacobian matrix at iteration *k*.

### Implementation

The implementation of the non-linear model proposed in this work is represented in the pseudocode shown in Algorithm 1 for a given load step at time level $t+\Delta t$. A load control-based algorithm is presented here. For each time step, the residual is solved for an incremental displacement. Creep, plastic and viscoplastic strains of shale, salt and sandstone rocks are computed in the material loop. The corresponding subroutines are represented by Algorithm 2 for salt rock, Algorithm 3 for shale rock and Algorithm 4 for sandstone. These algorithms are solved inside each element. Recalling the subdomains defined in Fig. 3, Perzyna’s approach (Algorithm 3) is applied for computing viscoplastic strains only in the subdomain $\Omega _\text {sh}$. Algorithm 4 goes over the sandstone elements belonging to $\Omega _\text {sd}$ for computing both creep and plastic (according to consistency approach) strains. Algorithm 5 is used for computing the increment of creep, and Algorithms 6 and 7 are used for computing the loading surface $p_y'$ and the hardening parameter $p_c'$ (yield surface), respectively. Notice in line 2 of Algorithm 3 that $\theta \leftarrow 0$, which means that the yield surface does not shrink during unloading, which is given by d$p_y'<0$ (see line 5 of Algorithm 7).
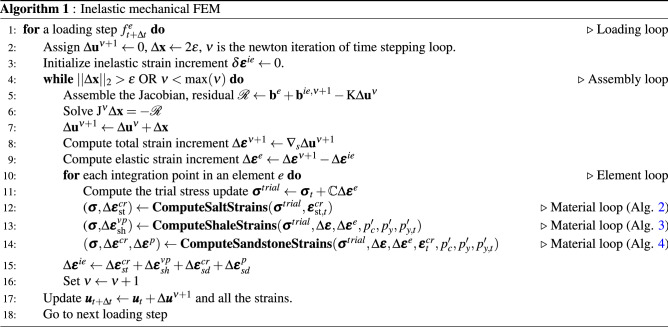

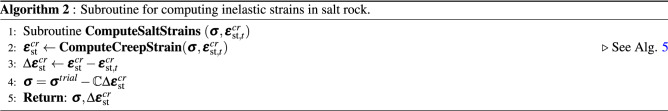

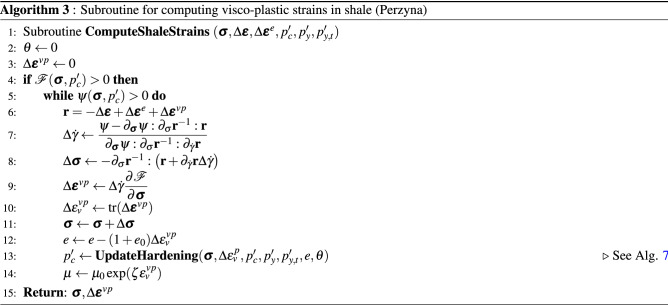

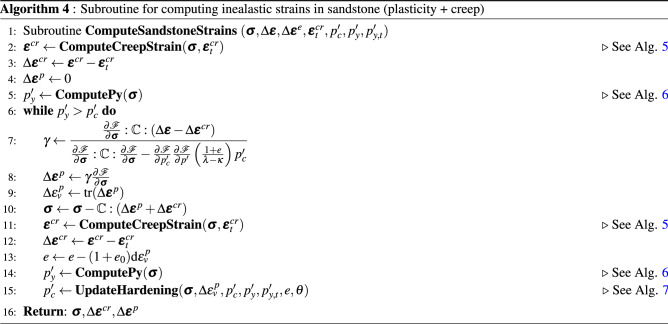

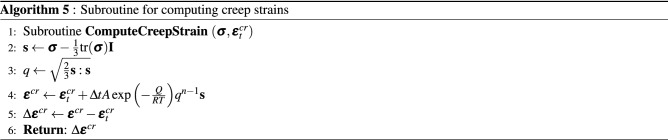

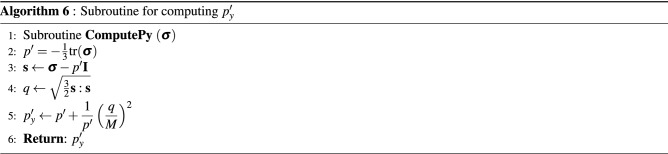

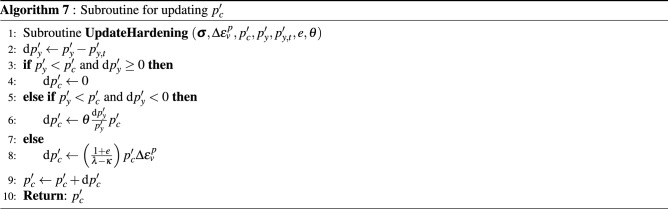


## Multiscale formulation

In the finite element formulation, the material properties (shear modulus, Poisson’s ratio, void ratio, etc) are defined for each grid element. Underground formations are often highly heterogeneous, with varying mechanical properties and rock types. This represents a serious challenge from a computational point of view since it requires extremely fine grids to appropriately capture all the heterogeneities, which naturally increases the computational effort for solving the resulting system of equations. To circumvent this problem we adopt a multiscale formulation, as proposed in^85^, in which the heterogeneities are properly represented in the fine scale but the system of equations is solved in a coarse scale, thus reducing computational burden while still capturing the physical characteristics of the system in a suitable manner.

**Figure 4:**
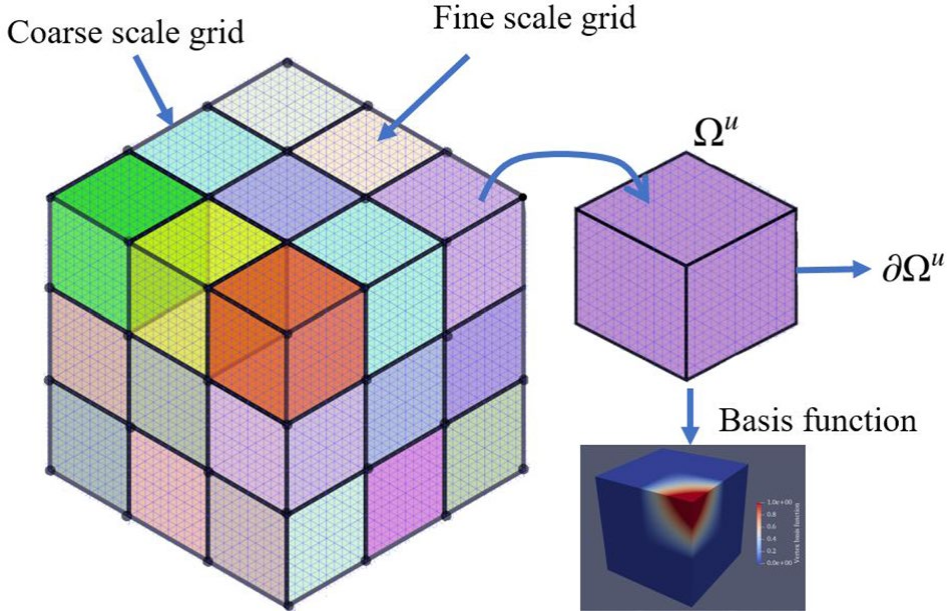
The above illustrates the 3D grid depicting finescale and coarse scale grid. Each coarse scale element ($\Omega _u$) consists of fine-scale elements. The basis function of the corner coarse element is shown.

The multiscale formulation is based on the construction of a coarse grid superimposed on a fine scale grid. Each element of the coarse grid contains a set of fine grid elements. The coarse elements are represented in Fig. 4 by the blocks with different colors. The goal is to compute the solution ${\textbf{x}}_H$ for the coarse scale and then project it onto the fine scale grid, ${\textbf{x}}_h$, that is,

49 $$\begin{aligned} {\textbf{x}}_h = P {\textbf{x}}_H \end{aligned}$$

where *P* is the prolongation matrix with dimensions $(n_h \times n_H)$, with $n_h$ and $n_H$ denoting the number of nodes in the fine and coarse scale grids, respectively. In addition, a restriction operator is defined as $R=P^T$, such that ${\textbf{x}}_H = R {\textbf{x}}_h$.

For the sake of generality, let us write the linearized system of Eq. (48) in the fine scale as

50 $$\begin{aligned} A_h {\textbf{x}}_h = {\textbf{b}}_h. \end{aligned}$$

The corresponding coefficient matrix and independent vector in the coarse scale can be obtained using the restriction and prolongation operators as $A_H = R A_h P$ and ${\textbf{b}}_H = R {\textbf{b}}_h$, respectively. Therefore, instead of solving Eq. (50), the coarse scale solution is obtained by solving

51 $$\begin{aligned} A_H {\textbf{x}}_H = {\textbf{b}}_H, \end{aligned}$$

which has $n_H$ unknowns instead of $n_h$. The solution is then prolonged to the fine scale as desired by using Eq. (49).

Clearly, the success of the multiscale strategy depends on the proper construction of the prolongation *P* and restriction *R* operators. These operators are actually constructed by computing displacement basis functions, ${\textbf{N}}_H$, at each element of the coarse grid. An example of a basis function is depicted in Fig. 4 for a three-dimensional element. For obtaining these basis functions, the following equations are solved for each coarse element

52 $$\begin{aligned} \nabla \cdot \left( {\mathbb {C}} : \nabla _s {\textbf{N}}_{H,i} \right)&= 0 \quad \quad \quad \text {in } \Omega ^u \end{aligned}$$

53 $$\begin{aligned} \nabla \cdot \left( {\mathbb {C}} : \nabla _s^{||} {\textbf{N}}_{H,i} \right)&= 0 \quad \quad \quad \text {in } \partial \Omega ^u \end{aligned}$$

54 $$\begin{aligned} {\textbf{N}}_{H,i} (x_j)&= \delta _{i,j} \quad \quad \quad \forall j \in [1, \dots , n_H^u] \end{aligned}$$

where $\Omega ^u$ and $\partial \Omega$ denote the domain of a given coarse element and its boundaries, respectively. The operator $\nabla _s^{||}$ is the symmetric nabla operator in the tangent plane of each face of $\partial \Omega$. Additionally, $n_H^u$ represents the number of fine scale nodes contained within the coarse element $\Omega ^u$.

The constitutive matrices in equation are defined for each element of the fine scale. In this manner, when the basis functions are obtained, they naturally take the internal heterogeneities into account. The system of equations represented by Eq. (54) is solved for each element of the coarse grid in the beginning of the simulation. A permutation matrix W as presented in^61^ is employed to rearrange all the interior (I), faces (F), edges (E) and vertex (V) nodes. The permuted system is written as,

55 $$\begin{aligned} {\hat{A}}_h = W^TA_hW = \begin{bmatrix} {\hat{A}}_{II} &{} {\hat{A}}_{IF} &{} {\hat{A}}_{IE} &{} {\hat{A}}_{IV} \\ {\hat{A}}_{FI} &{} {\hat{A}}_{FF} &{} {\hat{A}}_{FE} &{} {\hat{A}}_{FV} \\ {\hat{A}}_{EI} &{} {\hat{A}}_{EF} &{} {\hat{A}}_{EE} &{} {\hat{A}}_{EV} \\ {\hat{A}}_{VI} &{} {\hat{A}}_{VF} &{} {\hat{A}}_{VE} &{} {\hat{A}}_{VV} \\ \end{bmatrix}, \hat{u^h} = W^T\bar{u}^h = \begin{bmatrix} \hat{{\textbf {u}}}_I \\ \hat{{\textbf {u}}}_F \\ \hat{{\textbf {u}}}_E \\ \hat{{\textbf {u}}}_V \end{bmatrix}, \hat{f^h} = W^Tf^h = \begin{bmatrix} \hat{{\textbf {f}}}_I \\ \hat{{\textbf {f}}}_F \\ \hat{{\textbf {f}}}_E \\ \hat{{\textbf {f}}}_V \end{bmatrix}. \end{aligned}$$

By applying Gaussian elimination twice of the above system and solving for the reduced order boundary conditions as shown below,

56 $$\begin{aligned} {\widetilde{A}}_{FF} {\hat{u}}_{F} + {\widetilde{A}}_{FE} {\hat{u}}_{E} + {\widetilde{A}}_{FV} {\hat{u}}_{V}&= 0 \end{aligned}$$

57 $$\begin{aligned} {\widetilde{A}}_{EE} {\hat{u}}_{E} + {\widetilde{A}}_{EV} {\hat{u}}_{V}&= 0. \end{aligned}$$

where ${\widetilde{A}}_{FF},{\widetilde{A}}_{FE}, {\widetilde{A}}_{FV},{\widetilde{A}}_{EE},{\widetilde{A}}_{EV}$ is computed by solving the system of equations Eq. (54). The reduced upper triangle system is given by,

58 $$\begin{aligned} \begin{bmatrix} {\hat{A}}_{II} &{} {\hat{A}}_{IF} &{} {\hat{A}}_{IE} &{} {\hat{A}}_{IV} \\ 0 &{} {\widetilde{A}}_{FF} &{} {\widetilde{A}}_{FE} &{} {\widetilde{A}}_{FV} \\ 0 &{} 0 &{} {\widetilde{A}}_{EE} &{} {\widetilde{A}}_{EV} \\ 0 &{} 0 &{} 0 &{} {\hat{A}}_{VV} \\ \end{bmatrix} \begin{bmatrix} \hat{{\textbf {u}}}_I \\ \hat{{\textbf {u}}}_F \\ \hat{{\textbf {u}}}_E \\ \hat{{\textbf {u}}}_V \end{bmatrix} = \begin{bmatrix} \hat{{\textbf {f}}}_I \\ \hat{{\textbf {f}}}_F \\ \hat{{\textbf {f}}}_E \\ \hat{{\textbf {f}}}_V \end{bmatrix}. \end{aligned}$$

The ${\hat{A}}_{VV}$ is the coarse scale matrix ${\hat{A}}_H$ which is employed to solve for coarse scale solution. Further, this solution is prolonged to compute the finescale solution. In this work, this multiscale strategy is accommodated to solve for incremental loads which further helps in accounting the inelastic strains. Detailed algorithm is presented in Algorithm 8.
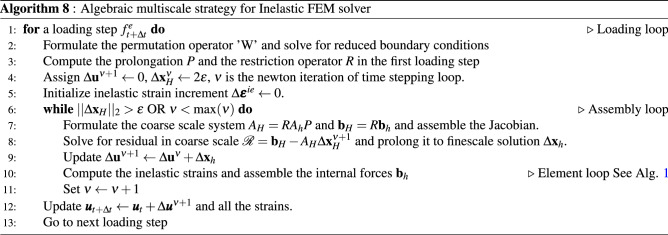


Depending on the required accuracy, the MS simulation method could be extended to iterative multiscale method (i-MS) using different preconditioners such as GMRES or ILU. The reader is referred to^85^ for a detailed description of how to employ iterative multiscale strategy.

## Results

Several numerical experiments are conducted to benchmark our unified framework to evaluate the impact of inelastic deformation at field scale when a cyclic load is employed. The consistency of the FEM formulation was checked and the displacement field was found to be 2nd order accurate in space.

### Benchmarking the simulator

**Figure 5:**
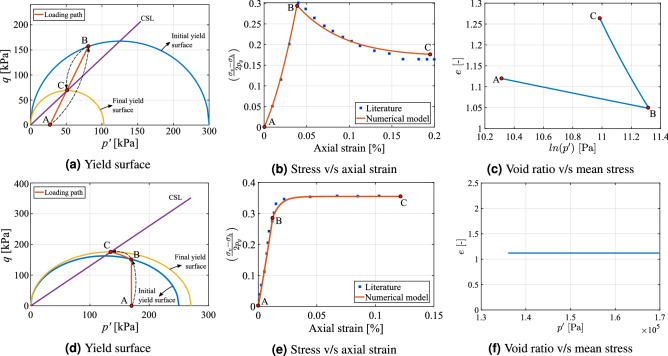
benchmarking: illustration of the strain softening scenario using MCC with consistency condition for drained rock (Fig. 5**a**–**c**) and strain hardening scenario for undrained rock (Fig. 5**d**–**f**) respectively. The simulation results are compared with^86^.

The simulator is benchmarked in the first step with the existing available data in the literature. In this test case, the material is assumed to be boston blue clay showing only plasticity without any creep and modeled using original MCC using Eq. (13). Drained loading condition with strain softening and undrained condition with strain hardening are considered in this section to study the variation of von Mises stress with strain. Table 1 show the parameters employed in the following simulation. The effective stress paths applied can be observed in Fig. 5a,d for drained and undrained loading conditions, respectively. As shown in these two graphics, the stress path goes from point A to B and from B to C. An increasing load is subjected to the rock similar to an over-time rise in the stresses when a fluid is continuously injected into the reservoir.

**Table 1:** Parameters employed for triaxial loading for Boston blue clay^86^.

#	Value	#	Value	#	Value
$\kappa$	0.034 (–)	$p'$	0.25 (MPa)	$\nu$	0.3 (–)
$\lambda$	0.17 (–)	*M*	1.34 (–)	$e_0$	1.12 (–)

The results are illustrated in Fig. 5. Figure 5a–c show the yield surface, variation of normalized stress with axial strain and variation of void ratio with mean stress respectively for drained condition. Similarly Fig. 5d,e illustrate the same material, but under undrained loading condition. In the case of drained condition (Fig. 5a), the load increases from point A until it touches the initial yield surface in point B. When it returns to point C, because it is in the softening region (above the CSL), the variation in volumetric strain is negative, which makes the yield surface to shrink according to Eq. (13). On the other hand, when undrained condition is imposed (Fig. 5d), the point at which the stress state reaches the initial yield surface (point B) is below the CSL, that is, in the hardening region, where volumetric strain variation is positive. As a result, the yield envelope expands in the plasticity regime from point B to point C.

Variation of normalized stress (normalized with preconsolidation pressure) with respect to the axial strain for drained (Fig. 5b) and undrained (Fig. 5e) conditions are compared with the literature of numerical results^86^. The characteristic softening and hardening behaviors can be verified in these two figures, with the stress increasing after point B for Fig. 5e, and decreasing for Fig. 5b. As it can be seen from these graphics, the numerical results compare relatively well with the literature. The variation of void ratio with logarithm of volumetric stress is shown in Fig. 5c,f. The change in void ratio for a drained condition is significant in inelastic region (from point B to C) compared to elastic region. However the void ratio remains constant when the load is applied in an undrained fashion because only the pore pressure changes in the rock.

The parameters in Table 1 were employed to verify the implementation of our work. In the field scale scenario, these parameters are calibrated further based on the observed uplift by the GPS stations which is elaborated in the later section. In a field case storage scenario depending on the operating conditions and time instant the reservoir will be usually partially saturated, so porous rock might experience drained or undrained consolidation conditions. The results presented above show that the numerical scheme is able to adequately describe these two situations.

### Comparison between finescale and multiscale results

**Figure 6:**
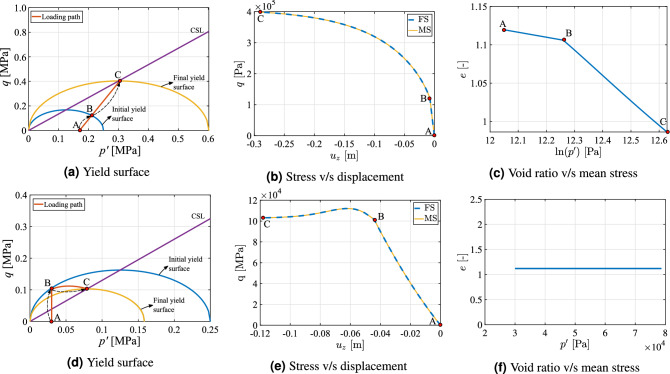
Comparison: illustration of the strain hardening scenario using MCC with consistency condition for drained condition (Fig. 6**a**–**c**) and strain softening scenario for undrained condition (Fig. 6**d**–**f**) respectively. Here the results are compared with FS and MS simulation methods.

In this test case, fine scale and multiscale results are compared for a homogenous rock using the same parameters as presented in Table 1. The respective stress paths for drained (Fig. 6a) and undrained (Fig. 6d) conditions are presented. In contrast to the test cases presented in the previous section, here the stress path for the drained condition promotes a hardening behavior (line B–C in Fig. 6a is below CSL), while softening is obtained for the undrained stress condition (line B–C is above CSL in Fig. 6d).

The results are presented in Fig. 6. Figure 6a–c show the yield surface, variation of normalized stress with axial strain and variation of void ratio with mean stress respectively for drained condition. Similarly Fig. 6d–f show the same for undrained condition. The variations of von Mises stress with axial displacement for finescale and multiscale are shown in Fig. 6b,e respectively for strain hardening and strain softening test case. The difference is virtually zero because the MS strategy is able to capture all the information from reduced order boundary conditions in the homogeneous rock. The variation of void ratio with logarithm of mean stress is shown in Fig. 6c,f. Similar to the previous test case, a higher change in void ratio is observed in the plastic region of the drained loading condition compared to the change in void ratio in the elastic region (that is from point B to C in Fig. 6c). In addition, the volumetric strain variation is zero for undrained loading, thus resulting in no change in void ratio.

### Cyclic loading of rocks

**Figure 7:**
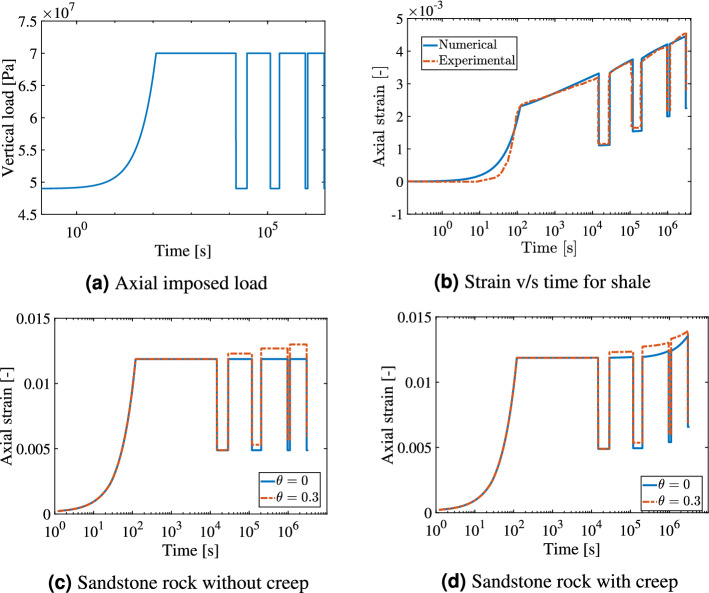
Cyclic loading: illustration of the effect of the imposed cyclic load (Fig. 7**a**) on shale rock and sandstone rock is presented here. The model parameters are chosen from the literature^46^ and compared with the experimental data^41^ as shown in Fig. 7**(b)**. Figure 7**(c**,**d)** show the variation of axial strain for sandstone rock without and with creep deformation for the same imposed loading respectively.

Underground energy storage will involve cyclic loading on the reservoir depending on the demand and supply of the energy (for e.g. H2). For this reason, it is important to study the effect of cyclic loading of rocks involving inelastic strains. In this test case, homogeneous sandstone and shale rock are studied to understand the effect of viscoplasticity on shale rock and the effect of creep and plasticity on sandstone rock. Considering seasonal storage of hydrogen in reservoirs, four phases of storage cycle are incorporated as elaborated in^87^. These phases include injection phase, rest phase at high pressure, withdrawal phase and lastly rest phase at low pressure. The chosen parameters for shale rock are presented in Table 2 and are obtained from^46^. The rock is modelled with $20 \times 1 \times 20$ elements of dimensions $25 \times 25 \times 50$ (cm$^3$). Bottom face is fixed, vertical cyclic load as shown in Fig. 7a is imposed on the top face and rest of the faces have a confining stress of 40 MPa.

**Table 2:** Parameters employed in the cyclic loading for shale rock^46^.

#	Value	#	Value	#	Value
$\kappa$	8.86e−3	$\mu _0$	9.69e12 (Pa s)	$\zeta$	4.33e3
$\lambda$	29.3e−3	*M*	1.3	$p_y$	43.17 (MPa)
Elements	$20 \times 1 \times 20$	*e*	0.089	Length	$25 \times 25 \times 50$ (cm$^3$)
A	1e−17	n	1.6	*Q*	5000 (mol/cal)

The variation of axial strain with time for numerical and experimental results^41^ is obtained here by employing Perzyna viscoplastic formulation. Viscoplastic behavior of shale rocks can be clearly observed in this test case, where the rock deforms of time at a constant load as shown in Fig. 7b. As the number of cycles increases, the axial strain in the rock also increases with time. In this test case, the unloading effect of the hardening parameter as explained in Eq. (14), is not accounted since this effect was not observed experimentally ($\theta =0$).

Using the same load as shown in Fig. 7a, the effect of cyclic loading on sandstone rock is studied. The parameters used for sandstone are presented in Table 1. For comparison purposes, however, the pre-consolidation pressure is chosen to be $p_y = 43.17$ MPa which is same as the shale rock. Figure 7c shows the variation of strain with time for sandstone without incorporating creep strains. It can be seen that in Fig. 7c the strain rate is 0 for constant load, since plasticity is a time-independent phenomenon. However, as $\theta$ is larger than zero, the accumulated strain increases with time for every cycle. This is due to the reloading effect, which is accommodated in the hardening parameter. When a fluid is stored in the subsurface based on the demand and supply, the higher the number of cycles for the same operating pressures, the higher will be the accumulated strain.

The interplay between creep and plasticity in sandstone was also studied. The parameters chosen for creep formulation are presented in Table 1. Figure 7d shows the variation of strain with time for sandstone by also considering creep strains. In this case, it can be seen that for a constant load, the strain is increasing with time due to the accumulation of creep strain. With the inclusion of creep and the reloading effect, number of cycles and the time period of the imposed load will make a significant impact on the accumulated strain.

Comparing shale rock and sandstone, it can be seen that the axial strain is higher for sandstone compared to the shale rock. This is because of the different elastic and plastic properties of the rock.

### Effect of heterogeneity on multiscale

In this test case, the goal is to study the influence of heterogeneities in the mechanical response of materials. For this purpose, Perzyna’s formulation is employed to observe the difference between fine-scale (FS) and multi-scale (MS) results. The strategy consists of imposing a uniform distribution for one material property at a time while the others are kept constant. The values for the heterogeneous paramenters and the corresponding ranges of variation are shown in Table 3. The remaining parameters (including domain dimensions) are taken from Table 2. The loading condition is shown in Fig. 7a. The domain has $20 \times 1 \times 20$ cells, with a coarsening ratio of $5 \times 1 \times 5$

**Table 3:** Different parameters chosen to study their influence of heterogeneity.

#	Value	#	Value	#	Value
$\kappa$	(5 $\pm 3) \times 10^{-3}$	$\lambda$	(30 $\pm 20) \times 10^{-3}$	$\zeta$	(10 $\pm 3) \times 10^{3}$
$p_y$	(40 $\pm 10) \times 10^{6}$ Pa	$\phi$	0.1 ± 0.05	$\mu _0$	(45 $\pm 30) \times 10^{12}$ Pa s

**Figure 8:**
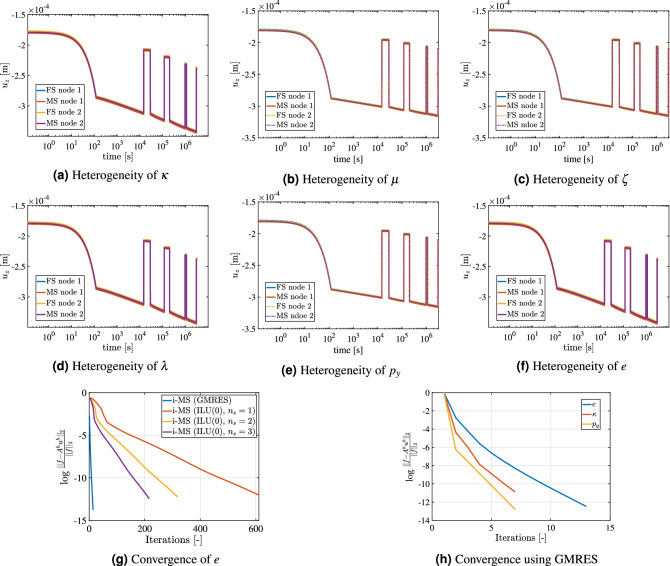
Heterogeneity: illustration of the impact of heterogeneity in shale properties on the axial deformation. The chosen parameters and its range is shown in Table 3. Heterogeneity in $\kappa$, $\mu$, $\zeta$, $\lambda$, $p_y$ and *e* is shown in the Fig. 8**(a**–**f)**, respectively. The convergence plot for void ratio is shown in Fig. 8**(g)** and the convergence plot for different parameter using GMRES is shown in Fig. 8**(h)**.

Figure 8 shows the comparison of FS and MS axial displacement results for two different nodes on the top face (node 1 and node 2). Among the investigated parameters, the deformation is most sensitive to $\kappa$ when heterogeneity is imposed: results obtained from two different nodes exhibit more pronounced deviations compared to the other scenarios. This occurs due to the influence of the parameter $\kappa$ on the bulk modulus which causes change in the elastic deformation. Due to this significant change, a slight difference of FS and MS results can be seen. Figure 8b–e respectively show the influence heterogeneity of $\mu$, $\zeta$, $\lambda$ and $p_y$ on the displacement over time. The impact of heterogeneity in void ratio Fig. 8f also affects the elastic part of deformation. However, the displacements at different nodes are not significant compared to Fig. 8a.

The results in Fig. 8 show that the MS solution is able to satisfactorily reproduce the FS solution. To achieve higher accuracies of MS solutions, an iterative MS strategy is employed. Figure 8g shows the variation of log($e_r$) with number of iterations. In iterative MS strategy^85^ there are two stages. The first stage involves the multiscale stage and the second stage involves the smoothing stage with a pre-conditioner. Depending on the required accuracy different numbers of smoothing stages $n_s$ can be applied. In this work, Incomplete LU decomposition (ILU(0)) with different number of smoothing stages and the Generalized minimal residual method (GMRES) are employed as a pre-conditioner. Figure 8g shows the convergence for each case. It can be seen that GMRES converges to the solution much faster in less than 10 iterations compared to ILU(0) as the pre-conditioner in multi-scale strategy. Figure 8h shows the convergence history for the GMRES as a smoother when heterogeneities in $\kappa$, *e* and $p_y$ are considered. It can be seen that it takes fewer iterations for heterogeneity in $p_y$ compared to the other parameters due to its influence primarily only on the inelastic deformation. MS results are quite satisfactory overall compared to the FS. In field scale situations, with high heterogeneities and lithological artifacts MS could vary from FS significantly.

### Field scale test case

Subsurface energy storage technology will involve cyclic injection and production in the reservoir, which can lead to subsidence/uplift. Due to these time-dependent processes, it is critical to understand the influence of inelastic strains and the stress distributions around the reservoir. To study this, a synthetic test case inspired from Bergermeer gas storage site in the Netherlands is chosen. Predominantly, it is a sandstone reservoir in the Slochteren formation located in the North West of the Netherlands with faults and fractures. The seal of this reservoir is predominantly a Zechstein salt layer (ZET). The other cap-rock seal is the Zechstein platten (ZEB) formation consisting of salt such as dolomite, anhydrites, clays and shale^11,88^.Due to the heterogeneous formation, the elastic properties and their plasticity constitutive laws vary accordingly. In this work the impact of faults and fractures are not considered in the geo-mechanical model. Although each layer is composed of different materials, for the sake of simplicity, it is assumed here a single type of material for each layer.

**Figure 9:**
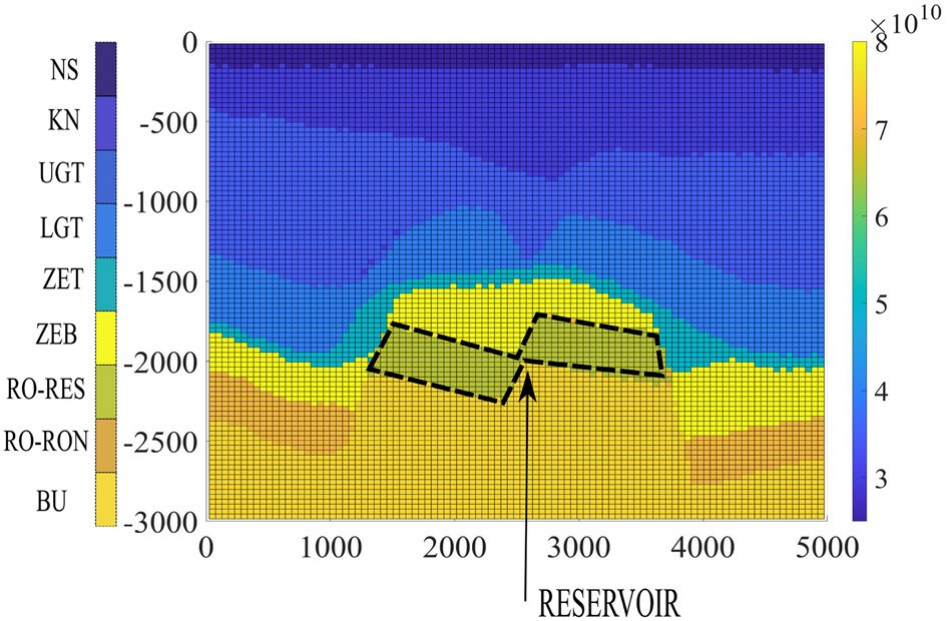
Field scale test case: the above shows the variation of the Youngs modulus inside the geological domain highlighting the reservoir boundary at a depth of around 2000 m. Roller boundary conditions are imposed in the left, bottom and right face and the top face has free stress boundary conditions.

A planar 2D cross-section of the reservoir is chosen^28^ as presented in Fig. 9 with varying Young’s modulus along the depth. The Young’s modulus and the Poisson’s ratio are chosen from the literature^89,90^. The domain has 98x1x98 cells, with the coarsening ratio of $14\times 1\times 14$ for the multiscale method. The properties chosen and the type of rock assumed in each layer is presented in Table 4. The choice of coarsening ratio has been extensively studied for simulation of multiphase flow^61,62^ based on CPU time for 3D test cases. In our study, we have taken approximately close to the root square of the elements existing in each axis. This is in agreement with the commonly used coarsening ratios, i.e., using the coarsening ratio of $10\times 10\times 10$ allows for reducing size of the mesh by 1 order of magnitude in each direction. The layers in the table are arranged along the depth in an increasing manner. Roller boundary conditions (zero normal displacement) are imposed on the left, bottom and right boundaries, whereas the top boundary is traction-free. In the earth’s natural state, lithostatic pressure would be present inside the geometrical domain. However, it is assumed that the lithostatic pressure does not cause nonlinear time-dependent deformation. The deviation from the equilibrium state, therefore, is only caused by injection and production of the reservoir, which is imposed as a uniform volumetric load over the elements in the reservoir. Figure 9 shows the area comprising the reservoir.

**Table 4:** Lithological properties in the Bergermeer field^89,90^.

Lithology	Young’s modulus (GPa)	$\nu$ (–)	Material
North Sea (NS)	25	0.25	Sandstone
Vlieland Claystone (KN)	30	0.25	Sandstone
Upper Germanic Triassic (UGT)	35	0.25	Sandstone
Lower Germanic Triassic (LGT)	40	0.25	Sandstone
Zechstein salt (ZET)	50	0.3	Rock salt
Zechstein Platten (ZEB)	80	0.25	Shale
Rotliegend Reservoir (RO-RES)	65	0.175	Sandstone
Rotliegend Non-Reservoir (RO-NON)	70	0.175	Sandstone
Carboniferous (BU)	75	0.2	Sandstone

**Table 5:** Parameters of plasticity, viscoplasticity and creep used for different rocks^44,46,79,86^.

Rock	Value	Value	Value	Value	Value	Value
Sandstone	$\kappa = 0.034$	$\lambda = 0.12$	$e_0=1.12$	$A = 1.5e$-30	$n =1.5$	$Q =5000$ (cal/mol)
Rocksalt	$A = 2.5e$-29	$n = 3.5$	$Q = 12325$ (cal/mol)			
Shale	$\kappa = 0.0076$	$\lambda = 8.19e$-3	$e_0=0.088$	$\zeta =4.33e5$	$\mu _0=9.68e12$ (Pa s)	

**Figure 10:**
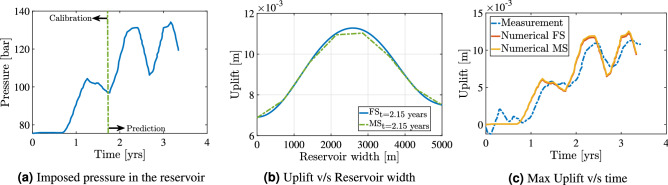
Field scale test case: the above illustration shows the results of uplift obtained from the reservoir model with inelasticity. Figure 10**(a)** shows the variation of imposed pressure with time indicating the calibation and prediction phases. Figure 10**(b**) shows the uplift computed at time = 2.15 years for both FS and MS methods. The variation of uplift with time is shown in Fig. 10**(c**) with the GPS measurement data^28,92^.

A number of researchers^28,91,93^ have tried to model the Bergermeer gas field by considering faults inside the 3D domain and employing the Kelvin Voigt model to account for inelasticity. However, in this work different inelastic formulations are employed for different materials to accommodate heterogeneities more effectively. Elasticity modulus degradation due to possible reservoir rock in-elasticity is neglected. The physical properties of all materials are considered to be isotropic. The material constants which are calibrated in the previous sections for lab-scale experiments cannot be used directly in the field scale measurements due to differences in stress magnitudes on the sample, the difference in the timescales of the lab experiments with the field scale experiments, and lastly the rock samples from different field can show different micro-structural properties. Hence in this work, the plasticity constants are calibrated based on the variation of the uplift with time in the period of 0 to 1.75 years. The parameters used in the inelasticity formulation are presented in Table 5. These parameters are calibrated based on the uplift data from the observed GPS stations. The calibration method can be further improved using optimization processes which is beyond the scope of this work. The results are presented in Fig. 10. The filtered uplift measurement data is obtained from GPS stations^28,92^.

Figure 10a shows the variation of the imposed pressure inside the reservoir to time. The calibration and prediction phase of the imposed pressure is separated at 1.75 years. Accordingly, based on the calibration the uplift predicted from our model is presented. Figure 10b shows the variation of uplift along the width of the reservoir at 2.15 years. The results show both fine scale and multiscale results. It can be seen that the uplift is not symmetric along the center of the reservoir, this is due to the asymmetrical position of the reservoir in the lithology. The multi-scale algorithm is able to compute the magnitude of the uplift quite satisfactorily when compared to the fine-scale solutions.

Lastly, Fig. 10c shows the variation of uplift with time for the imposed pressure loading. Here it can be seen that the measurement data from 0 to 0.8 years is not predicted from the numerical algorithm. This is because of the negligible change in the imposed pressure on the reservoir during this period. The next phase is the calibration phase from 0.8 years to 1.75 years. Here it can be seen that the magnitudes of the measurement data are close to the computed uplift, however, there is a lag between the measurement data and the numerical results. This lag can be due to (i) the poroelastic behavior of the reservoir which is not captured in this work, (ii) the well pressure is assumed to be the constant pressure acting on the entire reservoir as a body force, (iii) the effect of faults and fractures in the overburden causing a delay in the uplift, and (iv) lastly viscoelastic behavior of the rocks like sandstone and primary creep in rocksalt. There are evidences presented by the researchers of this delay in the measurement and the modelling results^17^. The visco-elastic effect and the damage of rocks effect is beyond the scope of this work. However, the magnitude of the uplift is satisfactory compared to the measurement data for both fine-scale and multiscale methods. The higher the magnitude of inelasticity of the rocks around the reservoir, the larger will be the uplift shown on the surface of the earth. The main emphasis of this test case, is to emphasise on the suitability of this computational framework to accommodate inelasticity effect of the rocks and showing the applicability of multiscale strategy in the field scale involving inelasticity.

In general, nonlinear mechanics simulations, specially with a multiscale framework, are expected to be sensitive to the mesh resolution; specially for heterogeneous media. The mesh sensitivity in our studies was limited, due to 3 main reasons: (1) very small incremental time steps (thus small loading steps) were imposed on our geo-models; (2) our multiscale strategy develops locally computed basis functions, capturing heterogeneities, without applying any upscaled parameter; (3) the development of an iterative multiscale procedure which guarantees systematic error control and reduction to any desired level. Note that the performance of the iterative strategy, specially its convergence rate, does depend on the size and geometry of the basis functions. Such performance study based on CPU time is outside the scope of the current manuscript.

This work provides the first of its kind attempt to match and predict the storage dynamics of the subsurface storage site, using the existing recorded sparse field data. Note that not all the degrees of freedom play equal role in the fitting procedure. More precisely, the stiff layers hardly contribute to the subsidence or uplift. Thus the main fitting sensitivities would lie in the soft sandtsone layers. It can also be observed that the material parameters calibrated for the Bergermeer test case are different from those calibrated in the previous test cases for lab experiments. This can be due to two reasons. First, the rock samples taken to the laboratory might not present the same type of grain structure as the in situ lithology even though they are same type of rock. Secondly, the operating conditions (loads and time-scales) in which the rocks are subjected in the field scale is different from the lab experiments. Therefore, more experimental data involving cyclic loading of rocks for longer time-scales are required for improving reliability of the numerical results.

**Figure 11:**
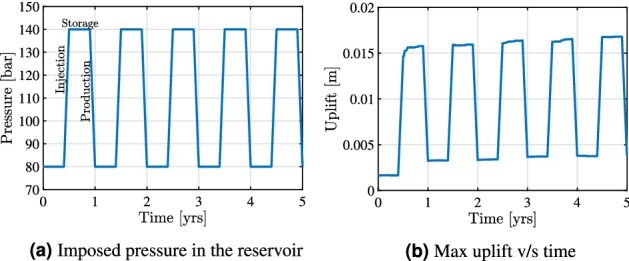
Field scale test case: the imposed pressure load is shown in Fig. 11**(a)**. Figure 11**(b)** shows the variation of the maximum uplift with time caused due to the imposed pressure.

The initial phase of testing the field site helped us identify the suitable pressures inside the field to operate feasibly. Using the minimum and maximum pressures inside the field as a reference, the Bergermeer site could be used as a storage site for energy. Every summer when there is excess renewable energy produced from solar and wind energy, the energy can be converted to green gases and stored, and accordingly, this energy could be used in the winter when there is high energy demand. Accordingly, to simulate these conditions, a similar pressure loading is applied as shown in Fig. 11a. For around half a year the energy is stored in the reservoir which implies a high pressure being imposed for roughly around six months and then the fluid is produced which reduces the pressure in the system close to its natural state of the reservoir.

Figure 11b shows the variation of the uplift concerning the time. Here we can see the accumulation of the uplift caused due to the inelasticity in the domain which is around 16 % (for 5 years) of the uplift caused due to the elasticity. Also, the amount of accumulated uplift in the first cycle is higher than the remaining cycles which is caused due to the plasticity of sandstone and shale that depend only on the imposed pressure. The remaining increase in the uplift with time is due to the creep deformation of the rocks in the rocks that is dependent on time. If we continue this operation for several decades, creep deformation might be more significant compared to the rest of the inelasticity effects. In this section, the change in inelasticity parameters caused due to prolonged loading is not considered which could be possible because of the change in the grain structure of the rocks. As the subsurface energy storage technology is growing because of the world drifting toward renewable energy, these reservoir sites can be used for multiple decades to store energy causing permanent uplift on the ground surface and causing damage to the living conditions of life.

## Conclusion

The main motivation of this work is to simulate and analyse the influence of inelasticity of porous rocks in subsurface formations relevant for cyclic energy storage. To study this, a computational framework is developed using an algebraic multiscale strategy to study the effects of plasticity, viscoplasticity and creep of different rocks found in the subsurface. Sandstone, shale and rock-salt rocks, which follow different physics and have different properties, are modelled using both fine-scale and multiscale methods using an implicit load driven time integration method. Depending on the required accuracy, a two stage iterative multiscale (MS) strategy was employed.

The simulator is benchmarked initially with the existing literature for different rocks. Comparison with the fine-scale solution, the multiscale results present acceptable accuracy with much lower computational cost. Subsurface storage technology involves cyclic loading over long periods. To study this effect, cyclic loading of shale and sandstone rocks is studied to understand the impact of inelasticity on strain with time. Lastly, a field test case is studied by including different types of rocks and their respective inelasticity physics. In this case, different lithological minerals were considered along with different elastic and plastic properties to model uplift on the surface level by using both fine-scale and multiscale methods. In this field test case, the uplift on the ground surface is compared with the computed uplift obtained from fine-scale and multiscale results.

The future scope involves accommodating visco-elasticity and further extending the formulation to poro-inelasticity to understand the effect of change in displacements on the pore pressure with time. Further, the computational framework should accommodate the modeling of faults and fractures in the field. This could be further used in modeling several porous fields realistically to understand the physics involved.

## Data Availability

The developed model is publicly available as open-source simulator in TU Delft repository of the ADMIRE project located at https://gitlab.tudelft.nl/ADMIRE_Public.
